# Remineralization of Artificially Demineralized Human Enamel and Dentin Samples by Zinc-Carbonate Hydroxyapatite Nanocrystals

**DOI:** 10.3390/ma15207173

**Published:** 2022-10-14

**Authors:** Stefan Kranz, Markus Heyder, Stephan Mueller, André Guellmar, Christoph Krafft, Sandor Nietzsche, Caroline Tschirpke, Volker Herold, Bernd Sigusch, Markus Reise

**Affiliations:** 1 Department of Conservative Dentistry and Periodontology, Jena University Hospital, Friedrich-Schiller University, An der alten Post 4, 07743 Jena, Germany; 2 Leibniz Institute of Photonic Technology (IPHT), 07745 Jena, Germany; 3 Center of Electron Microscopy, Jena University Hospital, Friedrich-Schiller University, 07743 Jena, Germany; 4 Otto Schott Institute of Materials Research, Friedrich-Schiller University, 07743 Jena, Germany

**Keywords:** enamel remineralization, dental erosion, biomimetic remineralization, dentin hypersensitivity, biorepair^®^, Raman spectroscopy, non-fluoride remineralization, dentinal tubules

## Abstract

(1) Background: Decalcified enamel and dentin surfaces can be regenerated with non-fluoride-containing biomimetic systems. This study aimed to investigate the effect of a zinc carbonate-hydroxyapatite-containing dentifrice on artificially demineralized enamel and dentin surfaces. (2) Methods: Human enamel and dentin discs were prepared and subjected to surface demineralization with 30% orthophosphoric acid for 60 s. Subsequently, in the test group (*n* = 20), the discs were treated three times a day for 3 min with a zinc carbonate-hydroxyapatite-containing toothpaste (biorepair^®^). Afterwards, all samples were gently rinsed with PBS (5 s) and stored in artificial saliva until next use. Samples from the control group (*n* = 20) received no dentifrice-treatment and were stored in artificial saliva, exclusively. After 15 days of daily treatment, specimens were subjected to Raman spectroscopy, energy-dispersive X-ray micro-analysis (EDX), white-light interferometry, and profilometry. (3) Results: Raman spectroscopy and white-light interferometry revealed no significant differences compared to the untreated controls. EDX analysis showed calcium phosphate and silicon dioxide precipitations on treated dentin samples. In addition, treated dentin surfaces showed significant reduced roughness values. (4) Conclusions: Treatment with biorepair^®^ did not affect enamel surfaces as proposed. Minor mineral precipitation and a reduction in surface roughness were detected among dentin surfaces only.

## 1. Introduction

Dental hard tissues such as enamel and dentin are constantly subjected to dynamic processes characterized by alternating periods of de-and remineralization. If there is a lack in remineralization, a loss in mineral content will occur. In addition to demineralization due to dental caries, tooth surface tissue can also be damaged by repeated consumption of acidic beverages and foods, also known as erosive tooth wear [1]. 

In particular, a loss in enamel will affect the tooth morphology, leading to dentin exposure followed by signs of hypersensitivity and functional impairment [2].

In this context, different remineralization strategies have been employed in order to regenerate initial hard tissue defects. 

For many years, the application of fluorides has proven to be an efficient measure which is still considered to be the gold standard in the prevention of dental caries and the treatment of early carious lesions. 

Fluorides affect the enamel by exchanging the hydroxyl group in hydroxyapatite to form either fluorapatite or fluor-hydroxyapatites [3,4]. Fluoride-substituted apatite is significantly more resilient towards acidic attacks [5]. During the nucleation process of partially dissolved minerals, fluoride ions will preferentially be included into the crystalline network, causing an increase in acidic resistance and remineralization speed [5,6,7,8]. Furthermore, topical application of fluorides (solutions, gels, varnishes) causes precipitation of calcium fluoride, not only on enamel, but also on exposed dentinal tubules, which is considered to be an efficient measure in the treatment of dentin hypersensitivity [9,10]. 

Besides fluoride modifying processes, non-fluoride enamel remineralizing systems have become popular, too. Currently, these are categorized into biomimetic systems and approaches that synergize fluoride efficacy [11]. In these terms, biomimetic strategies comprise all measures that involve the application of bioinspired materials that mimic natural remineralization of defective or diseased dental hard tissues [12].

In this context, the authors have already proven that application of casein-phosphopeptide-amorphous-calcium-phosphate enables efficient remineralization of artificially demineralized human enamel and dentin samples [13]. In a subsequent study, it was observed that the application of different biomimetic remineralization gels, rich in calcium and phosphates, caused the formation of a newly mineralized enamel-like layer on exposed dentin surfaces [14].

Contrary to fluoride-based remineralization, it was realized that the application of synthetic hydroxyapatite nanoparticles on decalcified enamel surfaces promoted the formation of organized hydroxyapatite surface crystals [15,16]. Moreover, it was found that synthetic hydroxyapatite crystals efficiently bind to enamel, acting as fillers of small defects and depression sites [11,17]. Furthermore, it was concluded that the bionic effect of nano-hydroxyapatite particles transmits to processes that mimic early enamel mineralization, due to increased concentrations of calcium and phosphate ions [15,18,19].

The resemblance of synthetic hydroxyapatite to natural enamel and dentine highlights its bioactive and non-toxic properties. Both micro- and nano-size hydroxyapatite are already used in oral care products, such as mouth rinses and toothpastes, with promising results on remineralization, biofilm management, caries prevention, dentine hypersensitivity, and teeth whitening [15,20,21,22]. 

Nevertheless, the efficiency of nano-hydroxyapatite-containing products in remineralizing decalcified enamel and dentin surfaces is still controversially discussed, especially when compared to the performance of fluorides [15,18,22,23,24].

Furthermore, the process of biomimetic remineralization on dental hard tissue using nano-hydroxyapatite crystals is still not fully understood. While some authors propose the formation of new synthetic enamel-like layers, others suggest that the applied crystals rather function as reservoirs for calcium and phosphate ions [11,17,18]. 

Therefore, the present in vitro study aimed to investigate the effects of repeated treatment with a zinc-carbonate nano-hydroxyapatite-containing dentifrice on the surface morphology of artificially demineralized human enamel and dentin samples. 

## 2. Materials and Methods

### 2.1. Sample Preparation

Forty extracted human third molars were collected and prepared for experimental use. Prior, the study was approved by a local ethics committee (ID-Nr.: 2019-1401_1-Material; Ethics Committee Friedrich-Schiller-University, Medical Faculty, Bachstrasse 18, 07740 Jena, Germany). 

**Figure 1:**
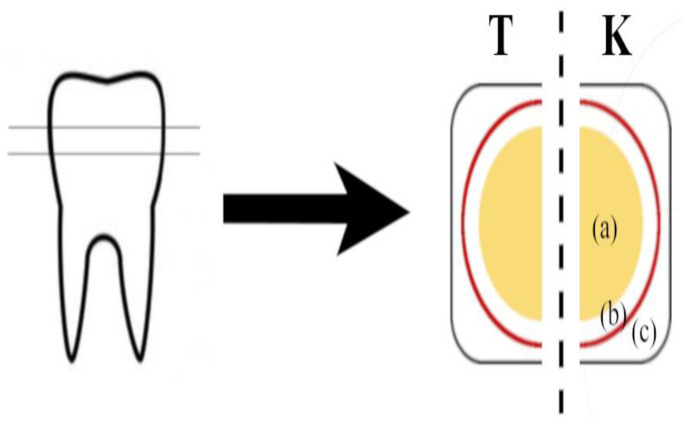
Preparation of enamel and dentin discs: (**T**) test group; (**K**) control group; (a) dentin; (b) enamel; (c) transparent epoxy resin.

After careful cleaning, the crowns were embedded in a cold-curing polymerizate (Kallocryl^®^, SPEIKO^®^, Bielefeld, Germany) and horizontally cut twice to receive flat enamel and dentin discs of 2 mm in thickness. The surface of the disc was then ground (#4000, waterproof silicon carbide paper; Struers, Copenhagen, Denmark) and subsequently divided into two pieces (test sample and control sample). For machine handling, all samples were further embedded into transparent epoxy resin (Specifix20, Struers, Copenhagen, Denmark) in a round mold. The prepared samples were then subjected to ultrasonic cleaning for 10 min in a 50% ethanolic solution. After drying, the uncovered upper sides of the sample discs were exposed to 35% orthophosphoric acid (Vococid, VOCO GmbH, Cuxhaven, Germany) for 1 min. In total, 20 control and 20 test samples were prepared. Sample preparation is schematically shown in Figure 1.

### 2.2. Experimental Set Up

Samples of the test group (*n* = 20) were exposed to a zinc-substituted carbonate-hydroxyapatite-containing dentifrice (biorepair bioniq, Dr. Kurt Wolff GmbH & Co. KG, Bielefeld, Germany) three times a day (morning, mid-day, evening). For this purpose, a pea-size amount (app. 250 mg) [25] of dentifrice was applied on the surface of the sample disc and carefully worked in by rotating movements using a soft brush for 3 min. Subsequently, all treated surfaces were gently rinsed with PBS for 5 s and stored in artificial saliva until the next treatment session. The described procedure was repeated each day for a total study time of 15 days.

Controls (*n* = 20) were treated the same way, without applying dentifrice. During the entire study time, all samples were kept in a moistened chamber. 

### 2.3. Raman Spectroscopy

For excitation, a single mode diode laser (Xtra, TOPTICA Photonics AG, Munich, Germany) at 785 nm emission was used which was coupled to a Raman microscope (Microprobe) connected to a Raman spectrometer (RXN1). The instrument was calibrated using a Raman calibration accessory, according to the routines outlined in HoloSpec software, version 4.1.0.234 (all from Kaiser Optical System, Ann Arbor, MI, USA). The Raman signal was detected on a Peltier-cooled (−60 °C), back-illuminated, deep-depletion CCD chip (Andor, Belfast, UK).

The laser light (100 mW) was focused on the water-immersed sample surface using a 60×/NA 1.0 water immersion objective (Nikon, Tokyo, Japan). 

For cross-sectional observation, lines of 41 Raman spectra, and for axial observation, lines of 42 Raman spectra, were obtained over the spectral region of 200 to 3550 cm^−1^ at a spectral resolution of 4 cm^−1^ with a step size of 1 µm and 1 s exposure time per spectrum. For data analysis, the Holomap software, version 2.5.0.0 (Kaiser) that runs under Matlab (The Mathworks, Natick, MA, USA) was used. The MCR (multivariate curve resolution) algorithm decomposed the data sets into weights and vectors that represented the concentrations and spectral contributions, respectively, of hydroxyapatite, biorepair^®^, embedding material, and background. Furthermore, the full width at half-maximum (FWHM) of the hydroxyapatite band near 960 cm^−1^, and for biorepair^®^ at 1450 cm^−1^ and 1463 cm^−1^, were calculated.

In addition to cross-sectional and axial observations, Raman images were also obtained for selected samples from an area of 20 × 20 µm, with a total of 60 × 60 spectra over the spectral region of 200 to 1800 cm^−1^ at a spectral resolution of 4 cm^−1^, with a step size of 0.33 µm and 1 s exposure time per spectrum. For this purpose, a confocal Raman microspectrometer with a 785 nm single-mode excitation laser, 60×/NA 1.0 water immersion objective, spectrograph, and Peltier cooled (−60 °C) CCD detector (CRM 300, Witec, Ulm, Germany) was used. Raman images were analyzed with Cytospec software, version 1.4.02 (www.cytospec.com, last access 13 October 2022). 

### 2.4. Energy-Dispersive X-ray Micro-Analysis (EDX)

The samples used for EDX were air-dried, mounted on sample holders, and carbon-coated (10 nm) to prevent surface charging utilizing a CCU-010 sputter coater (safematic GmbH, Zizers, Switzerland). Afterwards, the samples were analyzed using a scanning electron microscope LEO-1450 (Zeiss NTS GmbH, Oberkochen, Germany) equipped with an EDX system Quantax 200 with X-Flash 5030 detector (Bruker AXS, Berlin, Germany). Three samples from each group were analyzed.

### 2.5. White-Light Interferometry (Optical 3D Profilometry)

Three-dimensional surface measurements were performed using the white-light interference microscope Talysurf CCI HD (AMETEK Taylor Hobson Ltd., Leicester, UK) equipped with a 50× objective lens (single measuring field 330 µm × 330 µm, lateral image resolution approx. 400 nm). The vertical resolution was in the sub-nanometer range.

For each sample, an area of 0.55 mm × 0.55 mm consisting of a matrix of 3 × 3 stitched single measuring fields was observed. Each measurement was repeated five times with a total of three specimens in each group.

The data were analyzed using the software TalyMap Platinum, version 6.2.6746 (Taylor Hobson Ltd., Leicester, UK). On the basis of the obtained three-dimensional data sets, profile sections of 5 mm length were computationally generated; thus, the Ra parameters were determined by applying a gaussian filter (cut-off) λc = 0.8 mm.

### 2.6. Profilometry of Dentin Surfaces (Mechanical Profilometry)

Dentin surface roughness was analyzed using a profilometer Hommel Tester T 1000 (Hommelwerke GmbH, Villingen-Schwenningen, Germany). The data were recorded and documented using EVOVIS, version 1.40.0.2 (JENOPTIK Industrial Metrology Germany GmbH, Villingen-Schwenningen, Germany). Surface roughness was evaluated for each sample over a distance of 1500 µm at a constant speed of 0.15 mm/s. Each measurement was repeated five times with three samples in each group.

### 2.7. Statistical Analysis

Statistical data analysis was conducted using SPSS 22 (SPSS Inc., Chicago, IL, USA). The mean and standard deviation were calculated for the full width at half-maximum (FWHM) of the Raman band at 960 cm^−^^1^. Significant differences between the test- and control groups were analyzed using a paired *t*-test. The level of significance was set to 5%.

## 3. Results

### 3.1. Raman Spectroscopy

**Figure 2:**
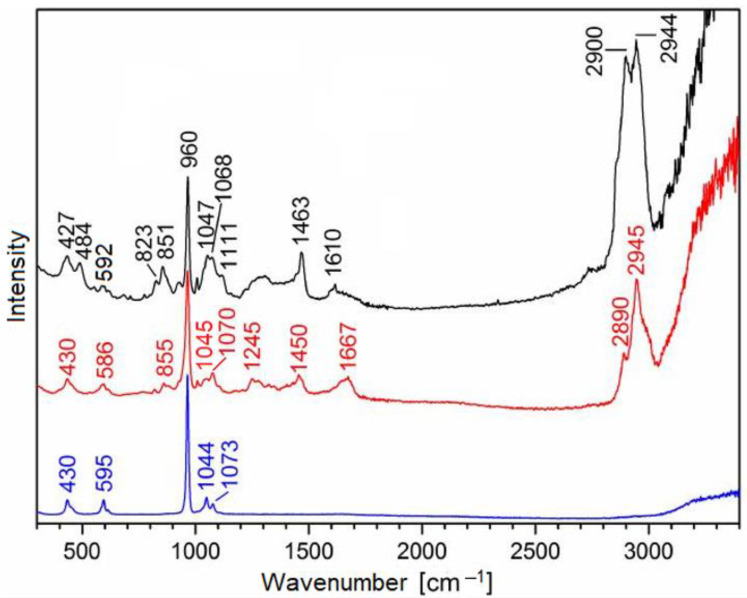
Raman spectra of biorepair^®^ (black), dentin (red), and enamel (blue).

Raman spectra obtained from biorepair^®^, dentin, and enamel are displayed in Figure 2. A summary of all Raman bands is shown in Table 1. Raman spectroscopy revealed the most intense band near 960 cm^−1^, which was assigned to the symmetric stretch vibration of PO_4_^3−^ in hydroxyapatite [26]. This band was present in enamel, dentin, and also biorepair^®^. In the dentin samples, a prominent band at 1450 cm^−1^ was assigned to the organic matrix, mainly collagen. A similar intense band at 1463 cm^−1^ was detected in the biorepair® sample, and was referred to as an organic additive. Further bands in the dentin samples at 1245, 1450, and, 1667 cm^−1^ were also assigned mainly to collagen. Bands at 427–430, 586–595, and 1044–1047 cm^−1^ were other modes of hydroxyapatite, while the bands at 1070–1073 cm^−1^ were referred to carbonate [14,26].

In addition, mineral crystallinity was determined from the obtained spectra. This feature was directly proportional to the inverse of full width at half-maximum (FWHM) of the Raman band at 960 cm^−^^1^ [26]. 

**Figure 3:**
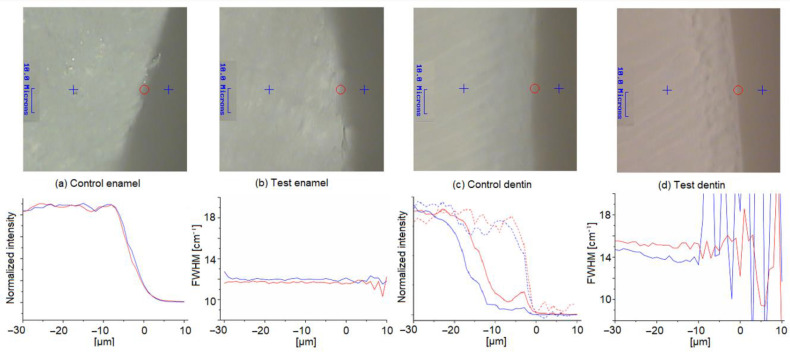
Micrographs of control and test samples with start, end, and reference points (upper row). Intensity of the hydroxyapatite band at 960 cm^−1^ and full width at half maximum (FWHM) for control (blue, solids) and test (red, solids) samples: (**a**) Intensity of the hydroxyapatite band at 960 cm^−1^ obtained from an enamel sample. (**b**) FWMH from enamel cross-sectional line profile. (**c**) Intensity of hydroxyapatite band at 960 cm^−1^ from dentin. Hydrocarbon band at 2900 cm^−1^ from the dentin control (blue, dashed) and dentin test sample (red, dashed). (**d**) FWHM from cross-sectional line profile obtained from dentin.

Representative Raman results of one dentin and one enamel sample are presented in Figure 3. A reference point was defined at the tooth margin. The start point for the line profile was set to a distance of 30 µm from the surface towards the inner proportion of the sample, while the end point was located 10 µm above the surface. The cross-sectional plots of intensities of the hydroxyapatite band and FWHM from one pair of test and control enamel samples are shown in Figure 3a,b. Between the enamel test and control samples, no significant changes were detected. In detail, the mean FWHM of the hydroxyapatite band in all enamel samples obtained by cross-sectional examination showed values of 11.64 ± 0.35 cm^−1^ for the test group and 11.52 ± 0.59 cm^−1^ for the control. 

The FWHM of biorepair^®^ was 13.14 ± 0.21 cm^−1^ with a 95%-confidence interval ranging between 12.72 and 13.56 cm^−1^. Both FWHM values of the hydroxyapatite band did not meet the confidence interval obtained for biorepair^®^. In addition, if a newly formed hydroxyapatite layer was present on the enamel surface, an elevated FWHM at the margin would have been expected. However, no significant differences between the test and control enamel samples were observed (*p* = 0.827). Furthermore, the existence of a biorepair^®^ layer would have been accompanied by elevated additive bands; e.g., at 1463 or 2900 cm^−1^. However, that was also not the case. 

The cross-sectional plots of intensities of the hydroxyapatite band and FWHM from one pair of test and control dentin samples are presented in the Figure 3c,d. In this regard, minor changes were found. In Figure 3c, the intensities of the hydrocarbon band at 2900 cm^−1^ from the organic matrix in dentin and from the putative organic additive of biorepair^®^ were additionally displayed. As can be seen, there is a gap between the hydroxyapatite and hydrocarbon distribution, which suggests an exposure of collagen fibers. This is probably due to the orthophosphoric acid-induced demineralization procedure. This is also faintly visible on the micrographs. 

The absence of a hydroxyapatite band at 960 cm^−1^ gives inconclusive and noisy FWHM values between position +10 and −10 µm that were therefore excluded from the calculations. A reduction in the gap and a relative increase in the hydroxyapatite content were observed for the evaluated test sample, but could not be confirmed by the other 19 samples. 

In regard to the dentin samples, a mean value of 13.52 ± 0.5 cm^−1^ was obtained for samples treated with biorepair^®^, which is within the range of the confidence interval. However, compared to the control group (FWHM 13.82 ± 0.87 cm^−1^), no significant difference was assessed (*p* = 0.311). The presence of a hydroxyapatite layer in dentin due to treatment with biorepair® would have been characterized by a decrease in FWHM at the margin, which was also not observed in the study.

**Figure 4:**
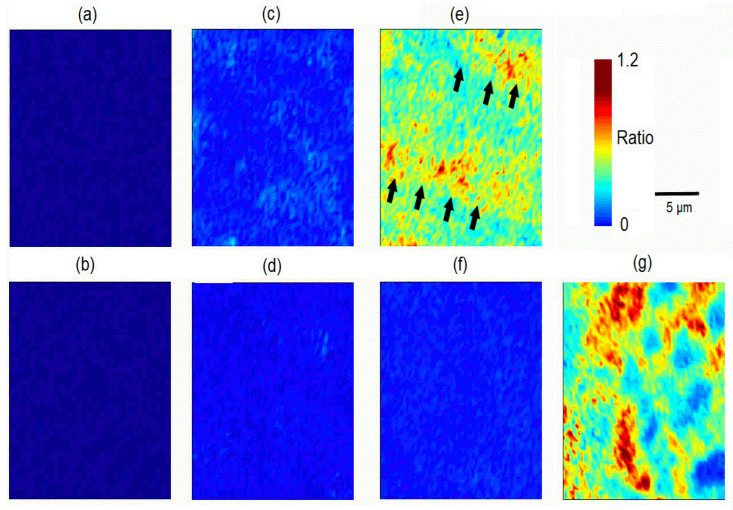
Color-scaled matrix-to-mineral ratios calculated from the Raman spectra at 960, 1450, and 1463 cm^−1^: (**a**) enamel test sample; (**b**) enamel control sample; (**c**) dentin test sample; (**d**) dentin control sample; (**e**) dentin test sample with high density bands marked by black arrow heads; (**f**) respective dentin control sample; (**g**) biorepair^®^ toothpaste aliquot.

Raman spectroscopic data also enabled the determination of the matrix-to-mineral ratio from the organic bands obtained from dentin (δ CH2; collagen) centered at 1450 cm^−1^ and biorepair^®^ at 1463 cm^−1^, and the most intense mineral band of hydroxyapatite near 960 cm^−1^. As the organic content is very low in enamel, no intensive Raman bands within the range of between 1430 and 1490 cm^−1^ were received. In this regard, a mean matrix-to-mineral ratio close to 0 was expected for enamel. In contrary, a ratio of 0.14 ± 0.04 was obtained for untreated dentin and 0.56 ± 0.1 for biorepair^®^. All values are presented as color-coded Raman images in Figure 4. 

Raman images did not reveal any signs of biorepair® deposition on the enamel test samples. The ratios in the dentin control (Figure 4d) were homogeneous and similar to those recorded for the enamel surfaces. The ratios in both test dentin surfaces were inhomogeneous, with some areas of higher values. For dentin treated with biorepair^®^ (Figure 4c), an average matrix-to-mineral ratio of 0.28 ± 0.21 was determined, which was slightly higher compared to the untreated dentin control (Figure 4d). In one observed dentin sample (Figure 4e), an even stronger intensity distribution was analyzed which differed from the respective control (Figure 4f), and looked similar to the intensity pattern observed for biorepair® with some patches of maximum ratio near 1.2 (Figure 4g). The detected intensity bands were of diagonal alignment (Figure 4e; black arrow heads).

### 3.2. Energy-Dispersive X-ray Micro-Analysis (EDX) 

**Figure 5:**
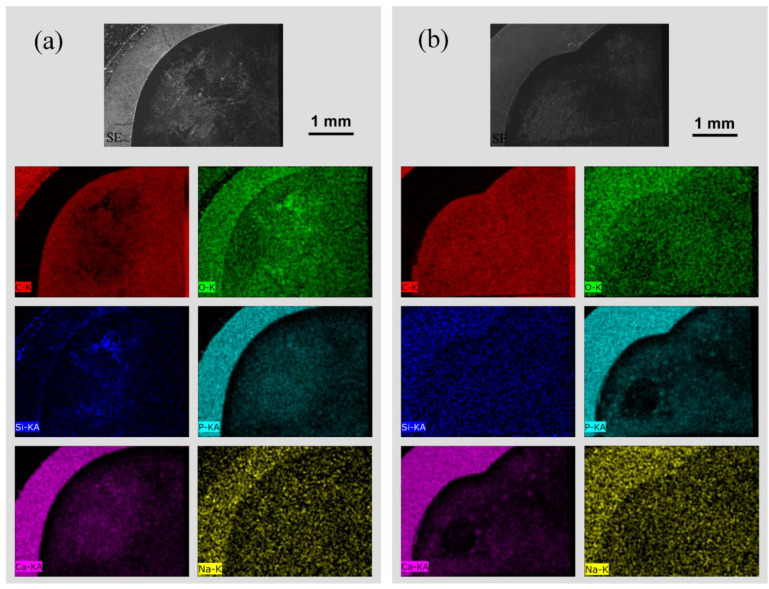
EDX analysis (mapping) of enamel and dentin samples: (**a**) test group treated with biorepair^®^; (**b**) non-treated control group. Top row shows SE-images of the enamel and dentin discs at 30× magnification. Analysis of C, O, Si, P, Ca, and Na are presented in different colors. Saturated areas within the dentin indicate intense deposition.

Results of the EDX analysis are shown in Figure 5. Samples from the dentin test group (Figure 5a) exhibited slight deposits of silicon and oxygen, and a homogeneous distribution of calcium and phosphorus on the surfaces. In contrary, evaluation of the non-treated dentin control samples did not deliver any extensive signals for Si, O, Ca, or P. Only minor traces of Ca, Cl, and P were present.

In the case of the enamel test and control samples, EDX-analysis did not reveal any accumulation of substances on the surfaces. 

### 3.3. White-Light Interferometry (Optical 3D-Profilometry)

**Figure 6:**
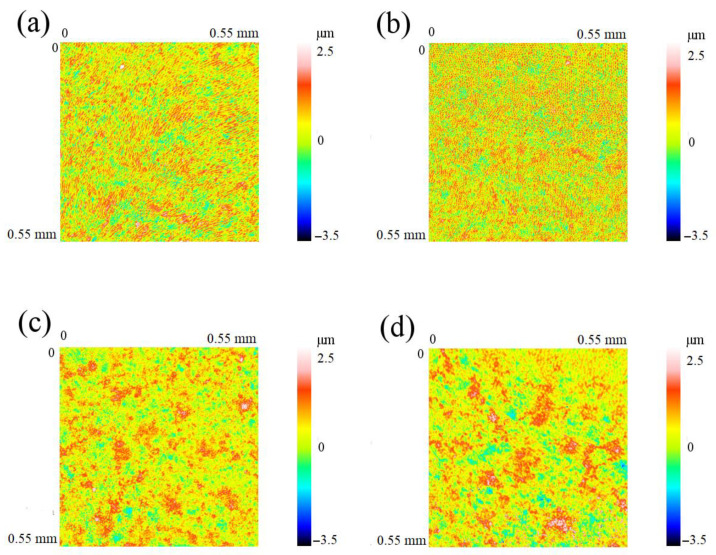
Results of the white-light interferometry (optical 3D profilometry) with color-coded representation of the surface topography: (**a**) enamel test sample; (**b**) enamel control sample; (**c**) dentin test sample; (**d**) dentin control.

White-light interferometric results of the optical 3D measurements were presented for test and control samples of enamel and dentin, with a color-coded representation of the surface topography (Figure 6). Two-dimensional Ra-values were determined along 5 mm traces. Mean Ra-values for the treated enamel surface were 0.58 ± 0.02 µm (Figure 6a). An identical result was obtained for the untreated enamel control samples (Figure 6b). Dentin treated with biorepair^®^ showed a mean Ra value of 0.62 ± 0.16 µm (Figure 6c), while the mean value of the dentin controls (Figure 6d) was analyzed with 0.51 ± 0.03 µm. A significant difference between the test and control samples was detectable in one sample that already showed an elevated matrix-to-mineral ratio in the Raman image (Figure 4e). Mean Ra values for the three samples are listed in Table 2. 

### 3.4. Profilometry of Dentin Surface (Mechanical Profilometry)

Evaluation of the dentin surface performed by profilometry showed mean Ra-values of 0.33 ± 0.05 µm for the test, and 0.46 ± 0.12 µm for the control samples. Pairwise comparison revealed significant differences (*p* < 0.0001). Results of the three tested samples are listed in Table 2.

## 4. Discussion

This study aimed to investigate the potential of a zinc carbonate-hydroxyapatite-containing dentifrice in remineralizing decalcified enamel and dentin surfaces. Although the overall effects were low, some changes, especially among the dentin samples, were observed.

Surface morphologic and topographic changes were analyzed by Raman-spectroscopy, EDX analysis, white light interferometry, and profilometry after 15 days of daily treatment. 

In past remineralizing studies, Raman spectroscopy has been applied by various authors in order to identify the type of newly formed surface minerals [27,28,29]. In accordance with results recently published by the authors of this study, an intense Raman band near 960 cm^−1^ was detected in the present study. This specific band can be assigned to the symmetric stretch vibration of PO_4_^3−^ in hydroxyapatite [26]. In the present investigation, the Raman band was found to be the key mineral signal in enamel, dentin, and biorepair^®^. 

Overall, Raman-spectroscopy revealed that treatment with biorepair^®^ does not result in the formation of a newly mineralized surface layer, as proposed [15,16]. 

In order to obtain more detailed information, mineral crystallinity was additionally analyzed by applying the full width at half maximum (FWHM) of the respective hydroxyapatite band near 960 cm^−1^ [14]. As suggested by Alebrahim et al. and references cited therein, mineral crystallinity is directly proportional to the inverse of FWHM of the Raman band at 960 cm^−1^, which means a narrow band width indicates high mineral crystallinity, while small bands are associated with low mineral crystallinity [26]. 

In the present investigation, an FWHM of 13.14 cm^−1^ was obtained for biorepair^®^. When compared to the FWHM of enamel (11.52 cm^−1^), the synthetic hydroxyapatite crystals in biorepair® are of significantly lower crystallinity. 

Treatment of enamel did not cause any significant change in the FWHM, which suggests that there is no effect of the biorepair^®^ treatment upon the mineral structure. In comparison, an FWHM of 13.52 cm^−1^ was observed for dentin samples treated with biorepair^®^, which is within the 95% confidence interval, but did not significantly differ from the untreated dentin control (FWHM 13.82 cm^−1^). In this regard, Raman spectroscopy did not reveal any significant changes in the mineral crystallinity of dentin surfaces treated with biorepair^®^. 

In contrast, a significant reduction in the FWHM of enamel and dentin was detected by the authors in a previous study, in which the efficiency of a biomimetic mineralization-kit (BIMIN) was evaluated [14]. The applied experimental kit was composed of an alkaline pretreatment solution containing calcium ions (pH = 9) and two different gelatin gels rich in phosphate, calcium, and fluoride ions. Constant treatment with BIMIN for 12 h resulted in a decrease in the FWHM from 16 towards 12.2 cm^−1^ in dentin, and from 12.5 to 12.4 cm^−1^ in enamel, which was associated with the formation of a newly mineralized surface layer [14]. 

In the present study, Raman spectroscopy did not reveal any such changes. Only in one treated dentin sample was an intensity pattern similar to that obtained for the biorepair^®^ aliquot, detected in one Raman image. This specific sample showed high intensity signals of diagonal alignment, which were concluded to be depositions of biorepair^®^ in longitudinally cut dentinal tubules.

The results of the present study are limited by the number of samples (*n* = 20) that were investigated, and by the study time (15 days). An increase in the number of samples and in treatment time would be a significant advantage. Optimizing the throughput of the instrumental approaches would also be beneficial, in order to receive larger data sets for a more thorough statistical analysis.

However, it has been shown that biorepair^®^ contains concentrations of 31.7 wt% in zinc-substituted hydroxyapatite [30]. In this context, Huang et al. observed that a concentration of 10% nano-hydroxyapatite is sufficient for remineralizing early enamel caries lesions. Lower concentrations will result in a significant drop in mineralization efficacy [31]. Furthermore, it was demonstrated that concentrations exceeding 15% nano-hydroxyapatite are not practical for usage in mouthwashes or toothpastes, because concentrations in this range inevitably generate some level of aggregation [18,31]. Because of this reason, zinc is often added, which provides some antibacterial activity, but also acts as an anti-calculus agent and crystallization inhibitor in toothpastes rich in nano-hydroxyapatite [15,32]. By adding Zn, hydroxyapatite concentrations of up to 31.7 wt% can be established in biorepair^®^ [30,33].

However, it has been hypothesized that zinc-carbonate hydroxyapatite nanocrystals are able to penetrate enamel pores, acting as templates during the mineral precipitation process. Furthermore, it was suggested that hydroxyapatite nanocrystals attract large amounts of Ca^2+^ and PO_4_^3−^ ions to the enamel surface that will occupy vacant positions in the crystalline apatite network [34]. 

However, because biologic hydroxyapatite minerals contain minor and trace elements, they are considered impure substances. The most important minor elements were found to be carbonates, magnesium, and sodium [35]. Detailed studies indicated that carbonate ions could be located in two anionic sites of the apatite structure: in PO_4_^3−^ sites (type B carbonated apatite) and in OH^−^ sites (type A carbonated apatite) [35]. Enamel consists mainly of B-type carbonated apatite, while dentin is of A–B mixed type (B > A) [36].

In the present study, precipitation of silicon and oxygen with a homogeneous distribution of calcium and phosphorus was detected on dentin surfaces that were treated with biorepair^®^. This is in line with a study performed by Bossú et al., who also observed an ensemble of C, O, Si, P, and Ca species solitarily on specimens that received treatment with biorepair^®^. Additionally, mineral penetration in the outmost enamel layer up to depths of 6.91 ± 0.92 µm, as well as biomimetic crystallization effects, were recognized in samples obtained from deciduous teeth [37]. 

Furthermore, it was shown by other authors that the application of synthetic hydroxyapatite crystals results in the formation of newly mineralized surface layers [15,31,38,39,40].

Poggio et al. concluded that the retention of calcium-phosphate minerals on tooth surfaces is an efficient measure in counteracting the effects of an erosive challenge. In this regard, the authors suggested that the mode of action is characterized by a combined effect of reduced demineralization and signs of remineralization/repair based on concentrations high in calcium and phosphate ions [41]. In contrast, a study performed by Ganss et al. suggested that products containing nano-zinc carbonate-hydroxyapatite, but no fluorides, are not efficient in reducing erosive tissue damage because synthetic zinc-carbonate-hydroxyapatite crystals quickly dissolve in acidic surroundings [42].

In the present study, changes in surface roughness were also observed among dentin samples that were treated with biorepair^®^. While white-light interferometry (optical 3D-measurements with a white light interference microscope) showed an elevated Ra value in one dentin sample (Ra 0.8 µm, Table 2) with dentifrice deposits in the Raman images, mechanical profilometry revealed reduced Ra values for dentin samples treated with biorepair^®^. The results are in line with a study performed by Chandru et al., who documented a reduction in surface roughness among specimens treated with biorepair^®^, too. Non-treated samples (controls) revealed uneven and rough surfaces with an increased porosity [34]. Furthermore, it was found that the application of nano-hydroxyapatite crystals results in a decrease in demineralization depth [43].

In summary, the present study revealed that biorepair^®^ treatment affects the surface of decalcified dental hard tissue only to a minor extent. Biorepair^®^ treatment for 15 days was not efficient in establishing a layer of newly-mineralized surface tissue. As proposed by other authors, the application of synthetic hydroxyapatite nano-particles should rather be seen as a supportive strategy in the process of demineralization/remineralization during an acidic challenge [15,17,41,42]. Therefore, assumptions that commercially available nano hydroxyapatite containing toothpastes are more efficient in remineralizing dental hard tissue defects as compared to fluorides should currently still be handled with care [15].

## 5. Conclusions

In the present study, the effect of a biomimetic dentifrice that contains nano-hydroxyapatite crystals (biorepair^®^) on artificially demineralized enamel and dentin samples was analyzed in regard to surface topographic changes. After treatment for 15 d, only minor effects on dentin surfaces were observed. While Raman spectroscopy did not reveal any significant changes, some precipitation of calcium-carbonate and silicon was observed by EDX analysis on dentin surfaces. In addition, a decrease in surface roughness was detected on dentin samples treated with biorepair^®^. In terms of enamel surfaces, no significant changes were observed. In this regard, any formation of newly-mineralized surface layers was not revealed. Whether remineralization of commercially available hydroxyapatite products is comparable to conventional treatment with fluorides needs still to be evaluated in detail.

## Figures and Tables

**Table 1:** Summary of all Raman bands with assignment and origin.

Raman Band [cm^−1^]	Assignment	Origin
427–430	δ_s_ PO_4_^3−^	biorepair^®^, enamel, dentin
484	additive	biorepair^®^
586–595	δ_as_ PO_4_^3−^	biorepair^®^enamel, dentin
851	additive	biorepair^®^
855	collagen	dentin
960	ν_s_ PO_4_^3^^−^	biorepair^®^, enamel, dentin
1044–1047	ν_as_ PO_4_^3^^−^	biorepair^®^, enamel, dentin
1068	additive	biorepair^®^
1070–1073	ν_s_ CO_3_^2−^	enamel, dentin, biorepair^®^
1245	amide III (collagen)	dentin
1450	δ CH_2_ (collagen)	dentin
1463	additive	biorepair^®^
1610	additive	biorepair^®^
1667	amide I (collagen), δ OH	dentin, H_2_O
2890–2900	ν_s_ CH_3_	biorepair^®^, dentin
2944–2945	ν_as_ CH_3_	biorepair^®^, dentin
3200–3400	ν OH	H_2_O

Stretch vibrations ν, deformation vibrations δ, symmetric s, antisymmetric as.

**Table 2:** Mean Ra values from three test and control samples obtained from optical and mechanical profilometry. Significant differences between the test and control groups are marked by (*).

White Light Interferometry Ra in µm (Optical Profilometry)				Profilometry Ra in µm (Mechanical Profilometry)	
Enamel		Dentin		Dentin	
test	control	Test	control	test	control
0.57	0.57	0.49	0.50	0.30	0.43
0.56	0.56	0.58	0.54	0.39	0.60
0.60	0.60	0.80	0.49	0.30	0.36
0.58 ± 0.02	0.58 ± 0.02	0.62 ± 0.16	0.51 ± 0.03 (*)	0.33 ± 0.05	0.46 ± 0.12 (*)

## Data Availability

Available upon request from the corresponding author.
